# Pre- and Peri-/Post-Compaction Follistatin Treatment Increases *In Vitro* Production of Cattle Embryos

**DOI:** 10.1371/journal.pone.0170808

**Published:** 2017-01-25

**Authors:** Guo Zhenhua, Sandeep K. Rajput, Joseph K. Folger, Liu Di, Jason G. Knott, George W. Smith

**Affiliations:** 1 Animal Husbandry Research Institute of Heilongjiang Academy of Agricultural Sciences (HAAS), Harbin, P.R. China; 2 Laboratory of Mammalian Reproductive Biology and Genomics, Michigan State University, East Lansing, MI, United States of America; 3 Department of Animal Science, Michigan State University, East Lansing, MI, United States of America; 4 Developmental Epigenetics Laboratory, Michigan State University, East Lansing, MI, United States of America; University of Florida, UNITED STATES

## Abstract

Our previous studies demonstrated that maternal (oocyte derived) follistatin (FST) expression is positively associated with bovine oocyte competence and exogenous follistatin treatment during the pre-compaction period of development (d 1–3 post insemination) is stimulatory to bovine early embryogenesis *in vitro* [blastocyst rates and cell numbers/allocation to trophectoderm (TE)]. In the present study, bovine embryos were treated with exogenous follistatin during d 1–3, d 4–7 and d 1–7 post insemination to test the hypothesis that embryotropic effects of exogenous follistatin are specific to the pre-compaction period (d 1–3) of early embryogenesis. Follistatin treatment during d 4–7 (peri-/post-compaction period) of embryo culture increased proportion of embryos reaching blastocyst and expanded blastocyst stage and total cell numbers compared to controls, but blastocyst rates and total cell numbers were lower than observed following d 1–3 (pre-compaction) follistatin treatment. Follistatin supplementation during d 1–7 of embryo culture increased development to blastocyst and expanded blastocyst stages and blastocyst total cell numbers compared to d 1–3 and d 4–7 follistatin treatment and untreated controls. A similar increase in blastocyst *CDX2* mRNA and protein (TE cell marker) was observed in response to d 1–3, d 4–7 and d 1–7 follistatin treatment. However, an elevation in blastocyst BMP4 protein (TE cell regulator) was observed in response to d 1–3 and d 1–7, but not d 4–7 (peri-/post-compaction) follistatin treatment. In summary, our study revealed the potential utility of follistatin treatment for increasing the success rate of *in vitro* embryo production in cattle. Such results also expand our understanding of the embryotropic actions of follistatin and demonstrate that follistatin actions on blastocyst development and cell allocation to the TE layer are not specific to the pre-compaction period.

## Introduction

Poor oocyte quality is recognized as a key limiting factor in improving the current low rate of successful assisted reproductive technologies and development of new options in infertility treatments. Over the last decades, understanding of the molecular determinants of oocyte quality has been a subject of intense study and still remains a major challenge in the field of reproductive biology especially in single ovulatory species. A large body of evidence suggests that maternal transcripts and proteins accumulated during oogenesis are crucial in determining competence of the oocyte to resume meiosis and develop into a viable embryo after fertilization [1–3].

Previously, our functional genomics studies in the bovine model revealed a characteristic RNA transcript profile of oocytes and adjacent cumulus cells associated with poor developmental competence [4, 5]. Of particular interest was increased transcript abundance of follistatin observed in good quality oocytes harvested from adult animals (control) compared to the poor quality oocytes obtained from prepubertal animals [4]. Moreover, maternal (oocyte-derived) follistatin mRNA and protein abundance was also greater in embryos that cleaved early and resulted in four times higher rates of blastocyst development compared to their late cleaving counterparts. Time to first cleavage is considered a reliable marker to select embryos with greater potential of implantation and successful pregnancy after transfer [3]. Hence, results suggested an association of maternal (oocyte-derived) follistatin with bovine oocyte competence.

Follistatin is a secreted glycoprotein first isolated from ovarian follicular fluid based on its ability to suppress follicle stimulating hormone secretion from pituitary cells [6]. Subsequently, FSH suppressing ability of follistatin was attributed to its high affinity binding and neutralizing ability for activin secreted from pituitary cells and therefore follistatin was classified as an activin binding protein [7]. Apart from activin, follistatin also can bind and regulate activity of multiple additional TGF-beta superfamily members such as inhibin and select BMPs [8–10]. Follistatin binding blocks the interaction of these ligands to their respective type I and type II serine threonine kinase receptors signaling through SMAD2/3 (activin, TGF-beta, nodal) or SMAD1/5 (BMPs) pathways [8, 11, 12]. Expression and functional role of multiple components of the TGF-beta superfamily including numerous ligands, receptors and their associated SMADs in oocytes and/or during embryo development suggest an important role of follistatin regulated TGF-beta signaling in early embryo development in cattle and human [13–16].

Given observed positive association of maternal follistatin abundance with oocyte and embryo developmental competence, we have previously investigated the functional role of follistatin in regulation of bovine early embryogenesis. siRNA mediated depletion of oocyte derived follistatin in zygotes reduced the number of embryos developing to the 8–16 cell stage and blastocyst stage and also decreased the number of total and TE cells in blastocysts [14]. Conversely, follistatin supplementation during the first 72 h (pre-compaction period) of bovine embryo culture (time window to embryonic genome activation and pre-compaction) increased number of early cleaving embryos, number of blastocysts, and improved the quality of resulting blastocysts by increasing their trophectoderm and total cell numbers. In addition, follistatin treatment also increased blastocyst mRNA abundance for the TE specific marker *CDX2* which is associated with increased pregnancy potential of blastocysts upon transfer [17]. Collectively, previous results demonstrated stimulatory effect of follistatin during bovine early embryogenesis *in vitro* when supplemented during the pre-compaction period (d 1–3) of embryo culture. Interestingly, follistatin mRNA is elevated in 16-cell embryos and detectable in bovine embryos through the blastocyst stage [4], suggestive of a potential regulatory role for follistatin during peri-/post-compaction (8–16 cell to d 7 Blastocyst) stages of embryo development. However, it is unknown whether this stimulatory effect is persistent or altered when exogenous follistatin is supplemented either during peri-/post-compaction period (d 4–7) or entire duration (d 1–7) of embryo culture *in vitro*.

Therefore, the present study was designed to investigate the potential effect of exogenous follistatin treatment during the peri-/post-compaction period (d 4–7) and entire duration (d 1–7) of *in vitro* embryo culture on promoting early embryonic development and quality at the blastocyst stage. We also examined whether the window of follistatin treatment had any effect on mRNA and protein abundance for select markers of blastocyst cell lineage determination.

## Materials and Methods

All chemicals and reagents were purchased from Sigma unless otherwise stated.

### In vitro oocyte maturation and fertilization and embryo production

*In vitro* maturation, fertilization and embryo culture to blastocyst stage were performed as described previously [18]. Briefly, cumulus-oocyte-complexes (COCs) were aspirated from antral follicles (3–7 mm in diameter) of bovine ovaries collected at a slaughterhouse. COCs containing homogenous oocyte cytoplasm and more than three layers of compact cumulus cells were matured in TCM 199 supplemented with 10% FBS (Hyclone, Logan UT), 5 IU/ml LH, 1 IU/ml FSH (Sioux Biochemical, Sioux Center, IA) and 1 μg/ml estradiol-17β at 38.5°C under 5% CO_2_ in air with maximum humidity. For *in vitro* fertilization (IVF), matured COCs were washed and co-incubated with motile spermatozoa (purified from frozen-thawed semen by Percoll gradient) for 20 h in fertilization medium at 38.5°C, 5% CO_2_ in air with maximum humidity. After IVF, presumptive zygotes were denuded from cumulus cells and associated spermatozoa by vortexing and then cultured from day 1–3 (pre-compaction period) in potassium simplex optimization medium (KSOM; EMD Millipore, Billerica MA) supplemented with 0.3% BSA. At 72 h (d3) post insemination (hpi), 8–16 cell embryos were separated, washed 3 times and cultured in fresh KSOM medium containing 0.3% BSA and 10% FBS from day 4–7 (peri-/post-compaction period).

### Follistatin treatment during pre-compaction and peri-/post-compaction period and effects on early embryonic development

**Fig 1:**
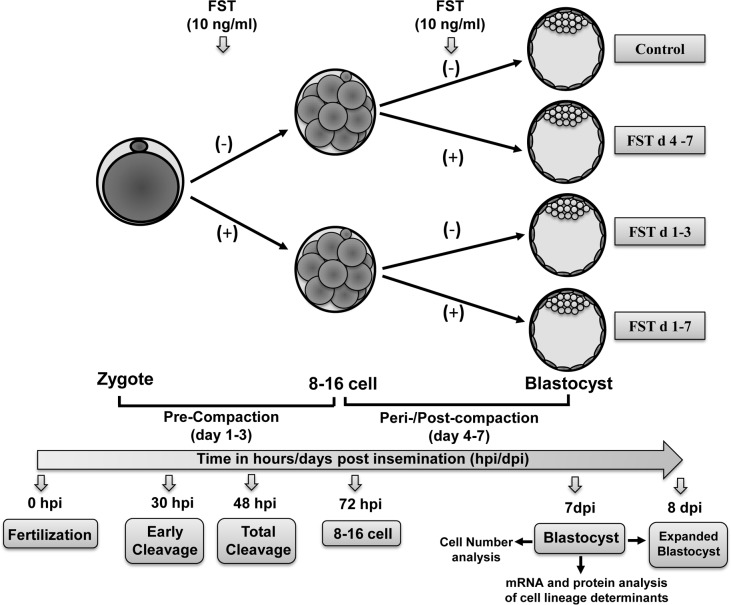
Experimental design of this study. After 20 hrs of fertilization, presumptive zygotes were cultured from day (d) 1–3 (pre-compaction period) in the presence and absence of 10 ng/ml follistatin (FST). At day 3, 8–16 cell embryos from control and follistatin treatment group were further cultured from day 4–7 (peri-/post-compaction period) in the absence and presence of follistatin (10 ng/ml). Early cleavage, total cleavage, 8–16 cell, d7 blastocyst and expanded blastocyst stages of pre-implantation embryo development were recorded at in all the treatment group including untreated control, FST d 4–7, FST d 1–3, and FST d1-7. Blastocysts from all the treatment groups were collected at day 7 and analyzed for cell number (total, TE, ICM) and mRNA and protein abundance for select markers/determinants of blastocyst cell lineage.

To determine whether positive effects of exogenous follistatin are specific to the pre-compaction period (d 1–3) of embryo culture, presumptive zygotes were washed and cultured to the blastocyst stage using four treatments: 1) untreated control embryos, 2) embryos treated with 10 ng/ml follistatin (maximal stimulatory dose; Lee et al., 2009) during d 1–3 (pre-compaction stage), 3) 10 ng/ml follistatin treatment during d 4–7 (peri-/post-compaction stage) and 4) 10 ng/ml follistatin treatment during d 1–7 (entire duration) of embryo culture *in vitro* (Fig 1). For all treatments, numbers of cleaved embryos were recorded at 30 h and 48 hpi to determine the follistatin treatment effect on proportion of early cleaving embryos and total cleavage rates respectively. In addition, proportion of embryos developing to the 8–16 cell, blastocyst and expanded blastocyst stages was also documented at 72 hpi, d 7 and d 8 of *in vitro* embryo culture respectively. All treatments were replicated 6 times using 50 zygotes per treatment. The blastocysts collected on d 7 were further analyzed to determine effects of treatments on total cell numbers (TCN) and allocation to trophectoderm (TE) vs. inner cell mass (ICM) cells using differential cell counting. A pool of 2–3 blastocysts from each treatment were used for real-time PCR analysis and a pool of 8–10 blastocysts from each treatment group were used for Western blot analysis to determine the effect of treatment on abundance of *CDX2*, *BMP4*, *TFAP2C* and *NANOG* transcripts (n = 6 replicates) and CDX2 and BMP4 protein (n = 4 replicates).

### Differential cell counting

Differential staining of inner cell mass (ICM) and trophectoderm (TE) was performed as described previously [14, 19]. Briefly, d7 blastocysts were exposed with 0.5% pronase in PBS for 3–5 min to remove zona pellucida and then washed in Hepes-buffered Tyrode’s solution (HbT). Zona free blastocysts were incubated in 1:5 diluted rabbit anti-pig whole serum (dialyzed in HbT) for 1 hour. Subsequently, embryos were washed three times for 5 min in HbT and placed in to 1:10 dilution of guinea pig complement for 1 hour containing propidium iodide and bisenzimide at final concentration of 10μg/ml. Embryos were then rinsed in HbT and analyzed under UV light for TE (red color) and ICM cell (blue color) counting using inverted fluorescence microscope.

### Quantitative real-time PCR

Total RNA isolation, cDNA synthesis and real-time PCR analysis of mRNA abundance in early embryos were performed as described previously (Patel et al., 2007; Bettegowda et al., 2007, 2008). All the primer sequences are listed in S1 Table. Relative abundance for target genes expression for each sample was estimated by 2^−ΔΔCt^ method as described elsewhere [20]. Amount of mRNA for target genes analyzed was normalized using ribosomal protein S18 (*RPS18)* expression as an endogenous control.

### Western blot analysis

Western blotting was performed using 10 blastocysts per lane lysed in 10 μl of RIPA lysis buffer (Sigma) containing 1X protease and phosphatase inhibitor. After mixing with an equal volume of 2X Laemmli buffer (Bio-Rad), samples were boiled for 10 min and cooled at room temperature for 5 min. The blastocyst lysates were then subjected to electrophoresis on 4–20% Mini-Protean TGX Precast gels (Bio-Rad) for separation of proteins and transferred to polyvinylidene fluoride membranes (Millipore). After transfer, membranes were incubated for 1 hr at room temperature in blocking buffer [1X TBST (Tris-buffered saline pH 7.4 with 0.1% Tween 20) with 3% BSA] followed by incubation with primary antibodies anti-CDX2 (Biogenex; #AM392) at 1:500, anti-BMP4 (Millipore; #MAB1049) at 1:1000 and anti-ACTIN (Millipore; #MAB1501) at 1:5000 dilutions in blocking buffer at 4°C overnight. Membranes were washed 3×5 min with 1X TBST and incubated with corresponding HRP-conjugated anti-rabbit-IgG (Cell Signaling Technology) or anti-mouse-IgG (Thermo Scientific) secondary antibodies at 1:5000 dilutions in blocking buffer for 1 h at room temperature. The immunoreactive protein bands were visualized by SuperSignal west Dura Chemiluminescent substrate (Thermo Scientific) and captured using myECL Imager (Thermo Scientific). Intensity of the individual bands was quantified using ImageJ densitometry software (http://imagej.nih.gov/ij/) and expressed relative to total ACTIN.

### Statistical analysis

Data were analyzed using one-way ANOVA. All percentage data from embryo culture experiments were arc-sine transformed prior to analysis. Fisher’s Protected Least Significant Difference (PLSD) test was used to detect differences in treatment means.

## Results

### Effect of window of follistatin treatment on preimplantation development

**Fig 2:**
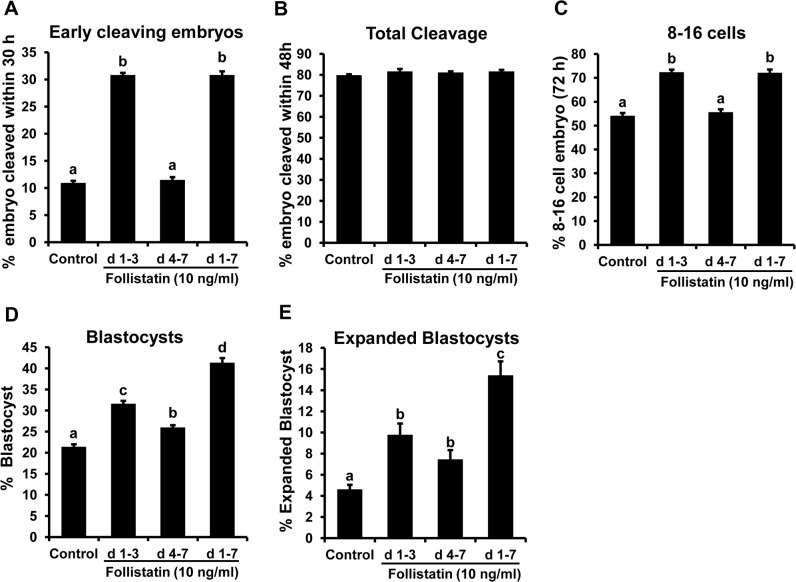
Stage-specific effects of follistatin treatment on bovine early embryo development. Effect of exogenous follistatin treatment during pre-compaction (d 1–3), peri-/post-compaction (d 4–7) and entire period (d 1–7) of *in vitro* embryo culture on; **(A)** First embryonic cleavage (30hpi). **(B)** Total cleavage rate (48hpi). **(C)** Proportion of embryos developing to the 8–16 cell stage on 3 d post insemination (pi). **(D)** Blastocyst rate (7 dpi). **(E)** Rate of expanded blastocysts (8 dpi). Values are shown as the mean ± SEM of the data collected from 6 replicates (n = 25–30 zygotes/treatment in each replicate). Values accompanied with different letters across the treatments indicate significant difference (P<0.05).

Effects of follistatin treatment during pre (d 1–3), peri-/post-compaction (d 4–7), and entire period (d 1–7) of *in vitro* embryo culture on indices of embryo development are shown in Fig 2. Follistatin supplementation during the entire period (d 1–7) of embryo culture resulted in a significantly higher (*P*<0.05) proportion of embryos reaching the blastocyst stage and undergoing expansion compared to d 1–3 and d 4–7 (Fig 2D and 2E) follistatin treatment and untreated controls. Embryos treated with follistatin during d 4–7 of embryo culture displayed increased (*P*< 0.05) percentage of embryos reaching blastocyst stage and undergoing expansion compared to the control group (Fig 2D). However, blastocyst rates for embryos treated with follistatin during the post compaction period (d 4–7) were lower than observed for embryos treated with follistatin during the pre-compaction period (d 1–3). As expected, follistatin treatment during d 1–3 of embryo culture increased early (Fig 2A) but not total cleavage (Fig 2B), and enhanced development to the 8–16 cell stage (Fig 2C) and blastocyst stage (Fig 2D), relative to untreated controls (Lee et al., 2009). The number of embryos that developed into expanded blastocysts following follistatin treatment during d 1–3 and d 4–7 of culture were also higher (*P*< 0.05) than untreated controls but not different between d 1–3 and d 4–7 follistatin treatment groups (Fig 2E).

### Effect of window of follistatin treatment on blastocyst cell allocation to ICM and TE

**Fig 3:**
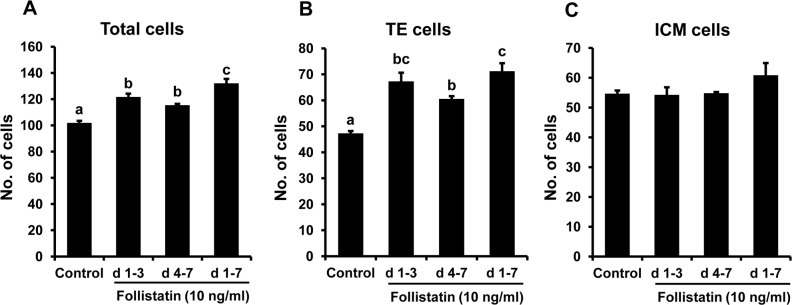
Stage-specific effects of follistatin treatment on bovine blastocyst cell allocation. Effect of exogenous follistatin supplementation during pre-compaction (d 1–3), peri-/post-compaction (d 4–7) and entire period (d 1–7) of *in vitro* embryo culture on; **(A)** Total cell number. **(B)** Number of TE cells and **(C)** number of ICM cells as determined after differential staining of resulting blastocyst on d 7 after insemination. Values are shown as the mean ± SEM of the data collected from 6 replicates (n = 25–30 zygotes/treatment in each replicate). Values accompanied with different letters across the treatments indicate significant difference (p<0.05).

As shown in Fig 3, blastocyst total and TE cell numbers were similarly enhanced (*P*< 0.05) following follistatin supplementation during peri-/post-compaction (d 4–7) or pre-compaction (d 1–3) periods of embryo culture when compared to untreated controls (Fig 3A and 3B). Follistatin treatment during the entire period (d 1–7) of embryo culture resulted in increased numbers of TE cells in resulting blastocysts relative to untreated controls and peri-/post-compaction follistatin treatment groups, but TE cell numbers were similar to the pre-compaction (d 1–3) follistatin treatment groups. Total cell numbers were maximal for the d 1–7 follistatin treatment group relative to other treatments. In contrast to blastocyst total and TE cells, the number of ICM cells in blastocysts remained unchanged in response to follistatin treatment provided during different time windows of embryo culture (Fig 3C).

### Effect of window of follistatin treatment on transcript and protein abundance of genes involved in blastocyst cell allocation

**Fig 4:**
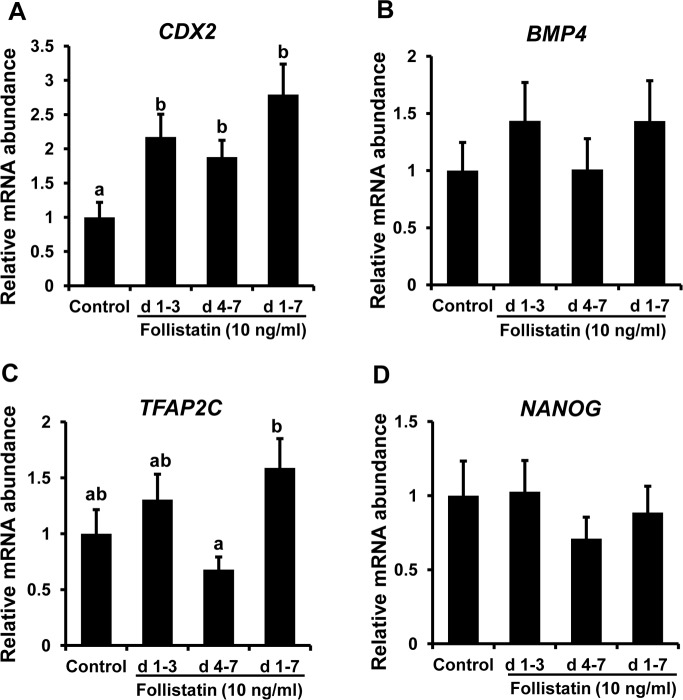
Stage-specific effects of follistatin treatment on mRNA expression of genes involved in TE cell lineage determination (*CDX2*, *TFAP2C and BMP4*) and ICM pluripotency (*NANOG*) in bovine d7 blastocysts. Effect of exogenous follistatin supplementation during pre-compaction (d 1–3), peri-/post-compaction (d 4–7) and entire period (d 1–7) of *in vitro* embryo culture on; **(A)** *CDX2*, **(B)** *BMP4*, **(C)** *TFAP2C* and **(D)** *NANOG* transcript abundance as determined by real-time PCR in bovine blastocysts collected on d 7 after insemination. Values are shown as the mean ± SEM of the data collected from 6 replicates (n = 25–30 zygotes/treatment in each replicate). Values accompanied with different letters across the treatments indicate significant difference (*P*<0.05).

**Fig 5:**
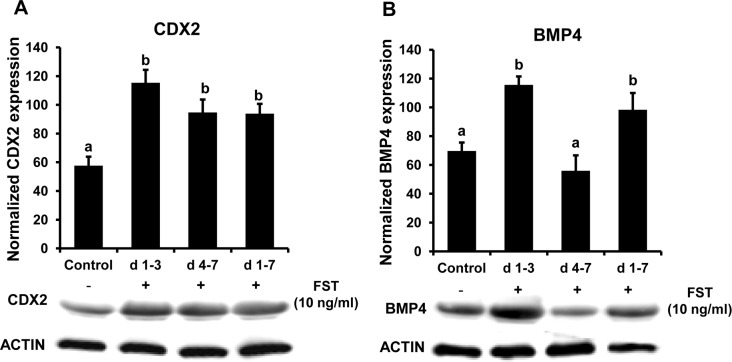
Stage-specific effects of follistatin treatment on protein abundance of TE cell lineage markers (CDX2 and BMP4) in bovine d7 blastocysts. Effect of exogenous follistatin supplementation during pre-compaction (d 1–3), peri-/post-compaction (d 4–7) and entire period (d 1–7) of *in vitro* embryo culture on **(A)** CDX2 and **(B)** BMP4 protein abundance as determined by Western blot analysis in bovine blastocysts collected on d 7 after insemination. Values are shown as the mean ± SEM of the data collected from 4 replicates (n = 10 blastocyst/treatment in each replicate). Values accompanied with different letters across the treatments indicate significant difference (*P*<0.05).

Real-time PCR analysis revealed that follistatin treatment (d 1–3, d 4–7 and d 1–7) increased (*P*< 0.05) the abundance of *CDX2* transcripts (Fig 4A), but did not change *BMP4*, *TFAP2C*, and *NANOG* transcript levels in d7 blastocysts, relative to untreated controls (Fig 4B–4D). Furthermore, Western blot analysis revealed that abundance of CDX2 was higher (*P*<0.05) in blastocysts from d 1–3, d 4–7 and d 1–7 follistatin treatment groups compared to the untreated controls (Fig 5A). Results also demonstrated higher (*P*<0.05) amounts of BMP4 protein in blastocysts from d 1–3 and d 1–7 follistatin treatment groups. However, blastocyst BMP4 protein abundance was not increased in response to follistatin treatment during d 4–7 (post compaction period) of embryo culture (Fig 5B).

## Discussion

Previous results from our laboratory demonstrated positive effects of follistatin treatment during the pre-compaction period on indices of embryo developmental progression [14]. Results from the present study revealed that (1). Embryotropic effects of exogenous follistatin treatment are not specific to exposure during the pre-compaction period (d 1–3), but are also observed when supplemented during d 4–7 of culture (after embryonic genome activation) (2). Early exposure (d1-3) to follistatin had some advantage over d 4–7 follistatin treatment as an increase in 8–16 cell embryos and blastocysts was observed (3). Embryotropic effects of follistatin on blastocyst rates and cell numbers (total, TE) were additive and maximal following follistatin exposure during the entire window of *in vitro* culture (d 1–7) (4). Elevated blastocyst BMP4 protein abundance was observed in response to follistatin treatment and response is stage dependent and not observed in response to d 4–7 (peri-/post-compaction treatment). While previous studies established a link between maternal (oocyte derived) follistatin and oocyte quality/embryo developmental progression [14], follistatin mRNA is detectable in bovine embryos from 1C stage to the blastocyst stage and elevated in 16-cell embryos [4]. This suggests that follistatin could have a potential regulatory role both during pre-compaction and peri-/post-compaction stages of embryo development. Collectively, these results support dynamic embryotropic actions of follistatin and TGF-beta superfamily signaling during multiple stages of bovine early embryo development.

In the present study, we demonstrated that effects of d 4–7 follistatin supplementation on blastocyst expansion and TE allocation are similar to d 1–3 follistatin treatment. As observed in the present study and also reported previously [14] d 1–3 follistatin treatment accelerated the time to first cleavage (within 30 hpi), suggesting that accelerated time to first cleavage in response to follistatin treatment may be linked to enhanced 8–16 cell embryo development and blastocyst rates. However, in the current study treatment with follistatin during d 4–7 of embryo culture also had a positive impact on blastocyst development, but developmental rates were slightly lower compared to embryos treated with follistatin on d 1–3. Results indicate embryotropic actions of follistatin are not specific to the pre-compaction period (i.e., d 1–3) of *in vitro* embryo development and are not be mediated solely by acceleration of time to first cleavage.

Follistatin is a TGF-beta superfamily growth factor binding protein that can bind and inhibit activity of activin and other TGF-beta superfamily members such as inhibin and select BMPs. Recent gene expression studies show that transcripts for select BMP ligands (BMP2, 3, 7, 10), type I and type II BMP receptors and their associated SMAD signaling proteins are elevated during the pre-compaction period but reduced after embryonic genome activation at 8–16 cell stage in cattle [15, 21]. In contrast, TGF-beta ligands such as BMP4 and BMP15 are prominently expressed during the post compaction periods (after embryonic genome activation) for both *in vivo* and *in vitro* produced bovine embryos [22]. Above results indicate that milieu of embryo derived TGF-beta growth factor superfamily ligands are dynamically regulated during pre- and peri-/post-compaction stages of bovine early embryonic development. Despite such potential differences in endogenous milieu of TGF-beta superfamily growth factors, similar effects of exogenous follistatin treatment during the pre- versus peri-/post-compaction period on indices of embryo developmental progression were observed and in general effects were additive. The exception to above is observed increase in blastocyst BMP4 protein in response to d 1–3 and d 1–7, but not d 4–7 follistatin exposure and hence is specific to the pre-compaction period. Collectively, results indicate that the targets of follistatin action and potential mechanisms linked to observed embryotropic effects may be different during pre- versus post-compaction periods of early embryonic development.

The mechanisms by which follistatin exerts its embryotropic effect during pre-compaction (d1-3, before EGA) treatment have recently been investigated in our lab. We observed that specific embryotropic actions of d 1–3 follistatin are ablated in the absence of SMAD4, a common SMAD required for TGF-beta superfamily signaling through SMAD2/3 (Activin, TGF-beta, Nodal) and SMAD1/5 (BMP) pathways [23]. Further inhibition of the SMAD2/3 signaling pathway using siRNA and a pharmacological approach caused a similar loss of d1-3 follistatin actions on embryo development [16]. This suggests that SMAD2/3 signaling is required for follistatin embryotropic functions during pre-compaction (d1-3) period. Follistatin effects were also observed during peri-/post-compaction period (d 4–7) of embryonic development which is comprised of several important stage-specific developmental events in cattle such as embryonic genome activation at 8–16 cell stage [24], polarization at 16-cell stage, [25] compaction at 32–64 cell stage [26] and TE cell lineage specification from d 5 morula to d 7 blastocysts stage [27]. Evidence suggests that TGF-beta signaling plays a key role in regulating genes associated with cell polarization, compaction, and blastocyst formation such as E-cadherin, fibronectin and Na+/K+ ATPase [28–31]. In addition, a recent study revealed that BMP signaling pathway is highly active during peri-/post-compaction period (after EGA) and required for cell cleavage in mouse preimplantation embryo development. Therefore, it is reasonable to speculate that d 4–7 follistatin embryotropic actions observed in the present study are mediated via modulating the endogenous growth factor milieu regulated genes involved in blastocyst development and may be linked to BMP signaling.

Given the higher number of TE and total cell numbers in follistatin treated blastocysts, we investigated if follistatin treatment altered the expression of genes involved in TE (*CDX2*) and ICM (*NANOG*) lineage determination. The transcript abundance of *NANOG* was not affected in response to follistatin treatment which was consistent with data that showed no significant change in ICM cell numbers in d7 blastocyst from follistatin treated embryos versus untreated controls. On the other hand, we observed that addition of follistatin to the culture media results in significant increase in *CDX2* mRNA and protein in blastocyst from d 1–3, d 4–7 and d 1–7 treatment groups. As a repressor of pluripotency regulatory networks and activator of trophoblast-specific genes, *CDX2* is a key regulator of trophoblast commitment in bovine embryos (Berg et al., 2011). In cattle, knockdown of *CDX2* mRNA using siRNA revealed a severe loss of the TE without any effect on ICM differentiation into the epiblast and hypoblast (Berg et al. 2011). These results suggest that follistatin mediated increased in *CDX2* transcription factor could affect the expression of genes involved in TE cell lineage determination in bovine embryos. However, the mechanisms by which follistatin exert this effect during pre-compaction (before EGA) and peri-/post-compaction period (after EGA) of embryonic development are yet to be explored.

Studies in mouse embryos and human embryonic stem cells (hESCs) showed that BMP signaling (SMAD1/5) plays an important role in TE lineage determination via regulating the expression of *CDX2* [32, 33]. In hESCs, higher amounts of autocrine BMP4 signaling increased *CDX2* expression and differentiation toward trophoblast lineage [33]. This mechanism may be conserved in cattle as *BMP4* and *BMP15* transcripts are prominently expressed during peri-/post compaction period of embryonic development [27]. In addition, we found that follistatin treatment during pre-compaction period (d 1–3) enhances BMP4 protein levels in d 7 embryos in the absence of further treatment (d 4–7) as indicated by increased BMP4 in blastocysts from d 1–3 and d 1–7 follistatin treatment groups, respectively. Hence, while it is plausible to speculate that the increase in CDX2 observed could be linked to follistatin induced increase in BMP4, increased CDX2 is also seen in d 4–7 follistatin treated embryos which do not display elevated BMP4 on d 7. Elucidation of the direct mechanism(s) of action and functional role of BMP4 in regulating CDX2 expression in bovine blastocysts will require further investigation.

In summary, results of the present studies demonstrate that embryotropic effects of exogenous follistatin on early embryogenesis are not limited to pre-compaction period but also manifest during peri/post-compaction period (d 4–7) of embryogenesis. Furthermore, the observed follistatin effects during d 1–3 and d 4–7 treatment are additive and further enhance blastocyst formation, expansion and total cell numbers when supplemented during the entire period (d 1–7) of embryo culture. These findings are critically important to understand the functional significance of follistatin in regulation of early embryogenesis in cattle and have direct translational relevance for increasing the efficiency of assisted reproductive technologies (ARTs) in dairy cattle. Apart from being a commercial livestock species, bovine preimplantation development is strikingly similar to human preimplantation development and the findings from our study may serve as a foundation for future clinical research in humans.

## Supporting Information

S1 Table Primers used for qRT-PCR. (DOCX)
