# Supplementary material for: Pre- and Peri-/Post-Compaction Follistatin Treatment Increases In Vitro Production of Cattle Embryos
Source: PLoS One. 2017 Jan 25;12(1):e0170808. doi: 10.1371/journal.pone.0170808 (PMC5266319; doi:10.1371/journal.pone.0170808)
Supplement: S1 Table — (DOCX) [file pone.0170808.s001.docx]

**S1 Table.** Primers used for qRT-PCR

| Gene name | Accession number | Primer Sequence (5' 3') |
| --- | --- | --- |
|  |  |  |
| *TFAP2C* | NM_001083748 | F: ATTCGCAAAGGTCCTATTTCCA  R: TAGATGTAGAGCTGAGGAGGGA |
|  |  |  |
| *BMP4* | XM_005211793 | F: CAAGCGTAGCCCCAAGCA  R: CGCCGGCAGTTCTTATTCTT |
|  |  |  |
| *CDX2* | AM293662 | F: FCGTCTGGAGCTGGAGAAGGA  R: CGGCCAGTTCGGCTTTC |
|  |  |  |
| *NANOG* | NM_001025344 | F: AAAGTTACGTGTCCTTGCAAACG  R: GAGGAGGGAAGAGGAGAGACAGT |
|  |  |  |
| *RPS18* | NM_001033614 | F: GTGGTGTTGAGGAAAGCAGACA  R: TGATCACACGTTCCACCTCATC |
|  |  |  |
